# Social Determinants of Health Among Those With and Without HIV Infection in NYC, the Epicenter of the U.S. Crisis

**DOI:** 10.1093/geroni/igab046.1639

**Published:** 2021-12-17

**Authors:** Tonya Taylor

**Affiliations:** SUNY Downstate Health Sciences University, Brooklyn, New York, United States

## Abstract

The COVID-19 pandemic in NYC, the epicenter of the US crisis, revealed indisputable evidence that social determinants of health (SDoH, e.g., racism, crowded housing, employment risks) and disparities in comorbid health risk factors produce higher burdens of disease and death among racial and ethnic populations. We conducted a needs assessment of SDoH among 1400 patients in several ambulatory care clinics to explore the impact among older adults, across different clinical populations. Among older adults with HIV (OAH), we found lower rates of food and housing insecurity compared to older adults without HIV. Despite higher levels of COVID knowledge and prevention adherence, we also found significantly higher levels of isolation, loneliness, depressive symptoms, and anxiety among OAHs compared to those without HIV. Access to Ryan White entitlements did buffer some impacts but preexisting high burdens of mental health issues were exacerbated, perhaps due to heightened perceptions of increased vulnerability to COVID-19.

